# Peritoneal Fluid from Patients with Ovarian Endometriosis Displays Immunosuppressive Potential and Stimulates Th2 Response

**DOI:** 10.3390/ijms22158134

**Published:** 2021-07-29

**Authors:** Joanna Olkowska-Truchanowicz, Agata Białoszewska, Aneta Zwierzchowska, Alicja Sztokfisz-Ignasiak, Izabela Janiuk, Filip Dąbrowski, Grażyna Korczak-Kowalska, Ewa Barcz, Katarzyna Bocian, Jacek Malejczyk

**Affiliations:** 1 Department of Transplantology and Central Tissue Bank, Centre of Biostructure Research, Medical University of Warsaw, 02-004 Warsaw, Poland; joanna.olkowska-truchanowicz@wum.edu.pl; 2 Department of Histology and Embryology, Centre of Biostructure Research, Medical University of Warsaw, 02-004 Warsaw, Poland; agata.bialoszewska@wum.edu.pl (A.B.); ala.sztokfisz@gmail.com (A.S.-I.); izabela.janiuk@wum.edu.pl (I.J.); 3 1st Department of Obstetrics and Gynecology, Medical University of Warsaw, 02-015 Warsaw, Poland; teksanskamasakra@o2.pl (A.Z.); fil.dabrowski@gmail.com (F.D.); ewa.barcz@interia.pl (E.B.); 4 Department of Obstetrics and Gynecology, Multidisciplinary Hospital Warsaw-Miedzylesie, 04-749 Warsaw, Poland; 5 Department of Gynecology and Obstetrics, Medical University of Silesia, 40-778 Katowice, Poland; 6 Department of Immunology, Faculty of Biology, University of Warsaw, 02-096 Warsaw, Poland; gkorczak-k@biol.uw.edu.pl; 7 Laboratory of Experimental Immunology, Military Institute of Hygiene and Epidemiology, 01-163 Warsaw, Poland

**Keywords:** endometriosis, peritoneal fluid, cytokines, chemokines, Th1 cells, Th2 cells, Treg cells, Th17 cells

## Abstract

Endometriosis is a common gynaecological disorder characterized by the ectopic growth of endometrial tissue outside the uterine cavity. It is associated with chronic pelvic inflammation and autoimmune reactivity manifesting by autoantibody production and abrogated cellular immune responses. Endometriotic peritoneal fluid contains various infiltrating leucocyte populations and a bulk of proinflammatory and immunoregulatory cytokines. However, the nature and significance of the peritoneal milieu in women with endometriosis still remains obscure. Therefore, the aim of the present study was to investigate the immunoregulatory activity of the peritoneal fluid (PF) from women with endometriosis. The peritoneal fluid samples were collected during laparoscopic surgery from 30 women with and without endometriosis. Immunoregulatory cytokines (IL-2, IL-4, IL-6, IL-10, IL-17A, IFN-γ and TNF) and chemokines (CCL2, CCL5, CXCL8 and CXCL9) were evaluated in PF and culture supernatants generated by unstimulated and CD3/CD28/IL-2-stimulated CD4^+^ T cells cultured in the presence of PF. The effect of PF on the generation of Treg and Th17 cells in CD4^+^ T cell cultures, as well as the natural cytotoxic activity of peripheral blood mononuclear cells, was also investigated. Concentrations of IL-6, IL-10, CCL2, CXCL8 and CXCL9 were significantly upregulated in the PF from women with endometriosis when compared to control women, whereas concentrations of other cytokines and chemokines were unaffected. The culturing of unstimulated and CD3/CD28/IL-2-stimulated CD4^+^ T cells in the presence of endometriotic PF resulted in the downregulation of their IL-2, IFN-γ, IL-17A and TNF production as compared to culture medium alone. On the other side, endometriotic PF significantly stimulated the production of IL-4 and IL-10. Endometriotic PF also stimulated the release of CCL2 and CXCL8, whereas the production of CCL5 and CXCL9 was downregulated. Endometriotic PF stimulated the generation of Treg cells and had an inhibitory effect on the generation of Th17 cells in cultures of CD4^+^ T cells. It also inhibited the NK cell cytotoxic activity of the peripheral blood lymphocytes. These results strongly imply that the PF from patients with endometriosis has immunoregulatory/immunosuppressive activity and shifts the Th1/Th2 cytokine balance toward the Th2 response, which may account for deviation of local and systemic immune responses. However, a similar trend, albeit not a statistically significant one, was also observed in case of PF from women without endometriosis, thus suggesting that peritoneal milieu may in general display some immunoregulatory/immunosuppressive properties. It should be stressed, however, that our present observations were made on a relatively small number of PF samples and further studies are needed to reveal possible mechanism(s) responsible for this phenomenon.

## 1. Introduction

Endometriosis is a common gynecological disorder affecting ca. 10% women of reproductive age. The disease is related to the endometrial-like tissue (endometrial glands and stroma) located outside the uterine cavity, mainly on the pelvic viscera and/or ovaries. Endometriosis is associated with chronic pelvic inflammation and manifests with dysmenorrhea, dyspareunia or chronic pelvic pain. It also accounts for ca. 50% of women’s infertility. Endometriosis is a debilitating disorder having a significant impact on patients’ quality of life. Nevertheless, the etiopathogenesis of this disease is still poorly understood [1,2,3,4].

There are several theories on the origin of the endometriosis; however, the most accepted cause of this disease is retrograde menstrual blood flow [5]. In this mechanism shed endometrial cells enter the peritoneal cavity, where they survive and form ectopic foci of the endometriotic tissue. This may be possible owing to the resistance of endometriotic cells to apoptosis and their increased adhesiveness and invasiveness [6,7,8,9]. It is also plausible that the formation of ectopic endometriotic lesions may also be facilitated by a permissive local peritoneal milieu as well as abrogated elimination of endometriotic cells by the cells of the local immune system, e.g., NK cells and macrophages [10,11].

Due to chronic pelvic inflammation and the elevated production of a variety of autoantibodies such as anti-nuclear, anti-phospholipid, and anti-endometrial antibodies, endometriosis may be considered as an autoimmune/autoinflammatory disorder [11,12,13,14]. The disease manifests with the local and systemic abnormal lymphocyte responses and abrogated NK cell cytotoxicity [10,13,15,16,17]. Pelvic inflammation includes peritoneal infiltration with a variety of immune cells including various subsets of lymphocytes, activated macrophages and granulocytes [18,19]. The endometriotic peritoneal milieu is also characterized by a local excessive production and accumulation of a bulk of proinflammatory and regulatory cytokines [20,21].

The role of the peritoneal milieu and the peritoneal fluid (PF) in the immunopathogenesis of endometriosis still remains obscure. Although endometriosis is considered to be an inflammatory disorder there is a growing body of evidence that the local peritoneal milieu may display, rather, an immunosuppressive character. Indeed, it has been reported that endometriosis is characterized by increased numbers of the peritoneal Treg cells displaying immunosuppressive and anti-inflammatory activity [22,23,24,25]. Furthermore, the PF from women with endometriosis also contains increased levels of suppressive anti-inflammatory cytokines such as TGF-β and IL-10 [20,26]. Thus, it is plausible that the PF from women with endometriosis may display some immunoregulatory properties. These properties, however, are still poorly characterized. Therefore, the present study was aimed at testing the immunomodulatory effects of the PF from women with endometriosis in comparison to control women without the disease. We investigated the effects of the PF on the immunoregulatory cytokine and chemokine production by the isolated CD4^+^ T cells as well as on the differentiation of CD4^+^ T cells into Treg and Th17 cells. Finally, we also tested the effect of PF on the cytotoxic activity of the peripheral blood natural killer (NK) cells.

## 2. Results

### 2.1. Concentrations of Cytokines and Chemokines in PF

Concentrations of IL-2, IFN-γ, IL-17A, TNF, IL-4, IL-10 and IL-6 in the peritoneal fluid of control women and patients with endometriosis are shown in Table 1. Women with endometriosis had increased concentrations of IL-6 and IL-10. There were no differences between the control and endometriosis groups in concentrations of IL-2, IFN-γ, IL-17A, TNF, and IL-4.

Concentrations of CCL2, CCL5, CXCL8 and CXCL9 in PF of control women and patients with endometriosis are shown in Table 2. PF from the women with endometriosis displayed significantly increased concentrations of CCL2, CXCL8 and CXCL9 as compared with the control subjects.

### 2.2. Effect of PF on Cytokine and Chemokine Production by CD4^+^ T Cells

**Figure 1:**
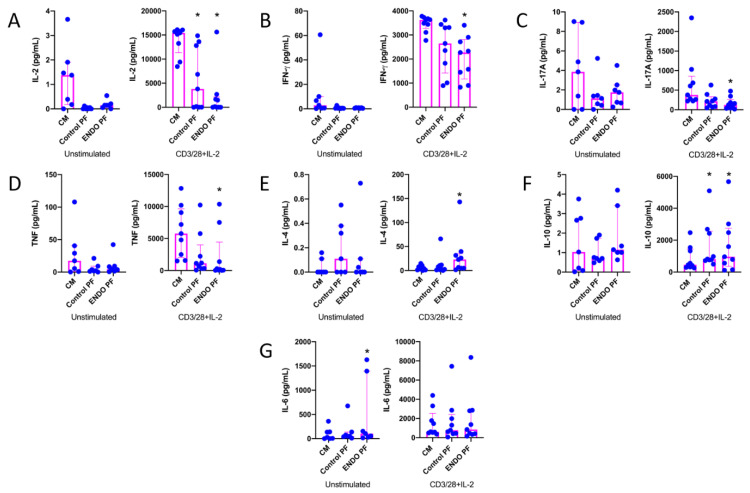
Production of (**A**) IL-2, (**B**) IFN-γ, (**C**) IL-17A, (**D**) TNF, (**E**) IL-4, (**F**) IL-10 and (**G**) IL-6 by cultured CD4+ T cells unstimulated or stimulated with CD3/CD28 beads and IL-2 in the presence of the culture medium alone (CM), peritoneal fluid from control woman (Control PF) or peritoneal fluid from woman with endometriosis (ENDO PF). The results are shown as scatter dot plots with a median and interquartile range. Statistical significance was computed by paired non-parametric ANOVA (Friedman’s test) followed by a post hoc test. * Statistically significant from the control group at least at *p* < 0.05. Baseline concentration ranges of the tested cytokines (pg/mL), respectively, in Control PF and ENDO PF used for the experiments were as follows. IL-2, 0.13–0.56 and 0.13–0.88; IFN-γ, 0–0.82 and 0–0.95; IL-17A, 0–8.10 and 0–8.70; TNF, 0.43–1.32 and 0.38–2.24; IL-4, 0–0.70 and 0–0.59; IL-10, 0.59–9.65 and 1.46–11.2; IL-6, 16.4–312.8 and 47.1–492.

To reveal whether the PF from the patients with endometriosis and the control subjects may affect the cytokine and chemokine production by CD4^+^ T cells, we evaluated cytokine and chemokine production following the 5-day culture of unstimulated and CD3/CD28/IL-2-stimulated CD4^+^ T cells, where stimulation with CD3/CD28 beads mimics antigen stimulation conditions [27,28]. The results of IL-2, IFN-γ, IL-17A, TNF, IL-4, IL-10 and IL-6 concentrations in CD4^+^ T cell culture supernatants are shown in Figure 1. As can be seen, CD4^+^ T cell stimulation with CD3/CD28 beads and IL-2 resulted in the very high upregulation of production of all tested cytokines as compared to unstimulated cells. The addition of the endometriotic PF to the culture of CD4^+^ T cells revealed its suppressive effect on the production of IL-2 (Figure 1A), IFN-γ (Figure 1B), IL-17A (Figure 1C) and TNF (Figure 1D), particularly by stimulated cells. Control PF also displayed some inhibitory activity toward the production of these cytokines; however, a statistically significant inhibition was seen only in the case of IL-2 production (Figure 1A). On the other hand, endometriotic PF significantly upregulated production of IL-4 (Figure 1E) and IL-10 (Figure 1F) by stimulated CD4^+^ T cells. The production of IL-10 by stimulated CD4^+^ T cells was also upregulated by the control PF (Figure 1F). The production of IL-6 by unstimulated CD4^+^ T cells was significantly stimulated by endometriotic PF, whereas the production of this cytokine by stimulated lymphocytes was affected by neither control nor endometriotic PFs (Figure 1G). There were no significant differences between PF from endometriosis and control group.

**Figure 2:**
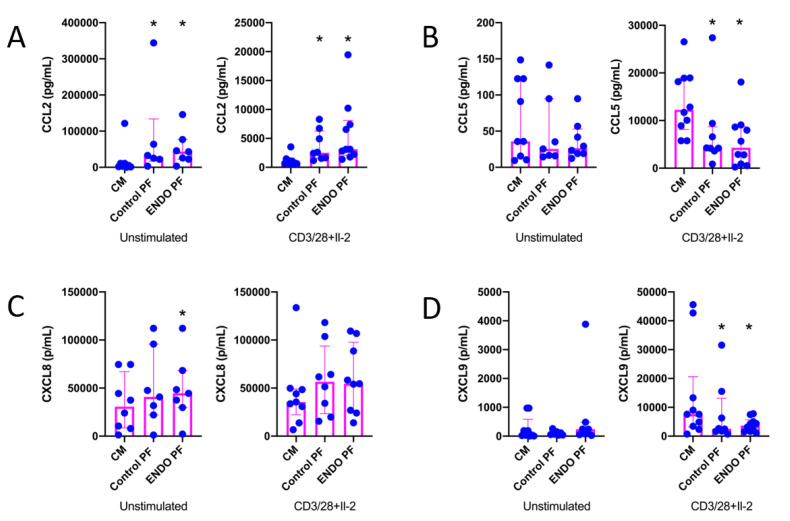
Production of (**A**) CCL2, (**B**) CCL5, (**C**) CXCL8 and (**D**) CXC9 by cultured CD4^+^ T cells unstimulated or stimulated with CD3/CD28 beads and IL-2 in the presence of the culture medium alone (CM), peritoneal fluid from control woman (Control PF) or peritoneal fluid from woman with endometriosis (ENDO PF). The results are shown as scatter dot plots with a median and interquartile range. Statistical significance was computed by paired non-parametric ANOVA (Friedman’s test) followed by a post hoc test. * Statistically significant from the control group at least at *p* < 0.05. Baseline concentration ranges of the tested chemokines (pg/mL), respectively, in Control PF and ENDO PF used for the experiments were as follows. CCL2, 16.6–198.7 and 10.12–393.2; CCL5, 2.5–9.6 and 6.0–33.9; CXCL8, 7.08–61.98 and 15.4–400.0; CXC9, 12.1–50.8 and 39.2–72.6.

The results of the evaluation of the effects of PF on chemokine (CCL2, CCL5, CXCL8 and CXCL9) production by CD4^+^ T cells are shown in Figure 2. As seen, CD3/CD28/IL-2 stimulation of CD4^+^ T cells significantly affected the production of all chemokines except CXCL8. It should be stressed, however, that production of the latter was extremely relatively high even in unstimulated CD4^+^ T cells. The production of CCL2 was significantly upregulated in both unstimulated and stimulated CD4^+^ T cells by the control as well as endometriotic PF (Figure 2A). The stimulation of CXCL8 production was seen only with endometriotic PF in unstimulated CD4^+^ T cells (Figure 2C). On the other hand, both control and endometriotic PF significantly inhibited the production of CCL5 (Figure 2B) and CXCL9 (Figure 2D) by stimulated CD4^+^ T cells. There were no significant differences between PF from endometriosis and control group.

### 2.3. Effect of PF on Generation of Treg and Th17 Cells

**Figure 3:**
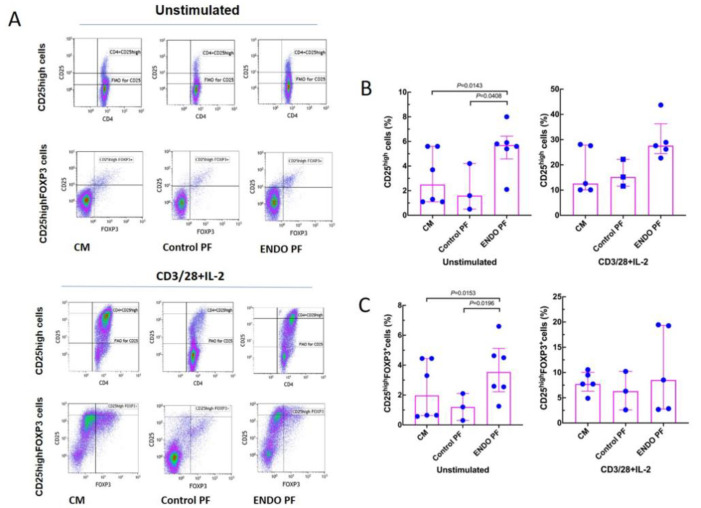
Effect of culture medium alone (CM), peritoneal fluid from control woman (Control PF) or peritoneal fluid from woman with endometriosis (ENDO PF) on generation of CD25^high^ and CD25^high^ FOXP3^+^ Treg cells in cultures of CD4^+^ T cells unstimulated or stimulated with CD3/CD28 beads and IL-2. (**A**) Gating strategy and a representative flow cytometry analysis showing identification of the respective CD25^high^ and CD25^high^ FOXP3^+^ T cell subpopulations in CD4^+^ T cells under different culture conditions. (**B**) Proportions of CD25^high^ T cells and (**C**) CD25^high^ FOXP3^+^ Treg cells in population of unstimulated or CD3/CD28 beads+IL-2-stimulated CD4^+^ T cells. The results are shown as scatter dot plots with a median and interquartile range. Statistical significance was computed by paired (Friedman’s test) or unpaired (Kruskal–Wallis test) non-parametric ANOVA followed by a post hoc test.

To see whether the PF from the patients with endometriosis and the control subjects may affect in vitro generation of Treg and Th17 cells we evaluated specific phenotype changes of the unstimulated and CD3/CD28/IL-2-stimulated CD4^+^ T cells following their 5-day culture. As seen in Figure 3, stimulation of CD4^+^ T cells with CD3/CD28 beads and IL-2 resulted in significant generation of CD25^high^ and CD25^high^ FOXP3^+^ Treg cells. The addition of the endometriotic PF significantly enhanced generation of the CD25^high^ T cells as compared to culture medium control and PF from women without endometriosis. A similar effect of the endometriotic PF on the generation of CD25^high^ FOXP3^+^ cells was also seen in cultures of unstimulated CD4^+^ T cells (Figure 3B), whereas there were no differences in the generation of CD25^high^ FOXP3^+^ cells in CD3/CD28/IL-2-stimulated cultures. Control PF did not affect generation of CD25^high^ and CD25^high^ FOXP3^+^ T cells in either unstimulated or stimulated CD4^+^ T cell cultures.

**Figure 4:**
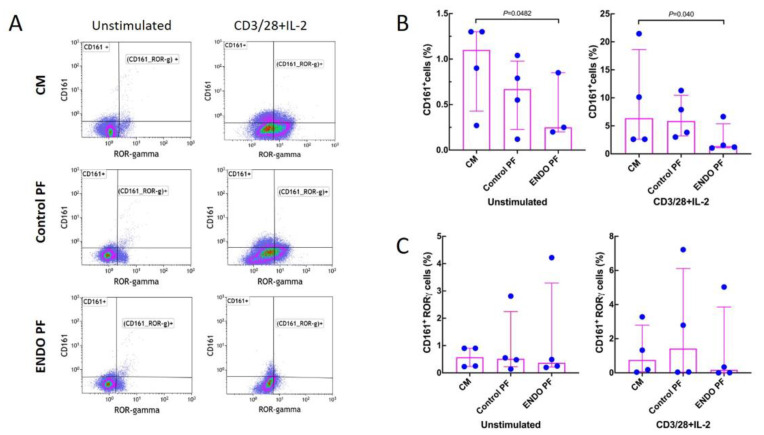
Effect of culture medium alone (CM), peritoneal fluid from control woman (Control PF) or peritoneal fluid from woman with endometriosis (ENDO PF) on generation of CD161^+^ and CD161^+^ RORγ^+^ Th17 cells in cultures of CD4^+^ T cells unstimulated or stimulated with CD3/CD28 beads and IL-2. (**A**) Gating strategy and a representative flow cytometry analysis showing identification of the respective CD161^+^ and CD161^+^ RORγ^+^ T cell subpopulations in CD4^+^ T cells under different culture conditions. (**B**) Proportions of CD161^+^ T cells and (**C**) CD161^+^ RORγ^+^ Th17 cells in population of unstimulated or CD3/CD28 beads+IL-2-stimulated CD4^+^ T cells. The results are shown as scatter dot plots with a median and interquartile range. Statistical significance was computed by paired (Friedman’s test) or unpaired (Kruskal–Wallis test) non-parametric ANOVA followed by a post hoc test.

Figure 4 shows that stimulation with CD3/CD28/IL-2 significantly increased the generation of CD161^+^ T cells while having no significant effect on the generation of the CD161^+^ RORγ^+^ cells. Endometriotic PF had a significant suppressive effect on generation of CD161^+^, both in unstimulated and stimulated CD4^+^ T cell populations (Figure 4A). Neither PF affected the generation of CD161^+^ RORγ^+^ cells (Figure 4B). There were no significant differences between PF from endometriosis and control group.

### 2.4. Effect of PF on NK Cell Cytotoxicity

**Figure 5:**
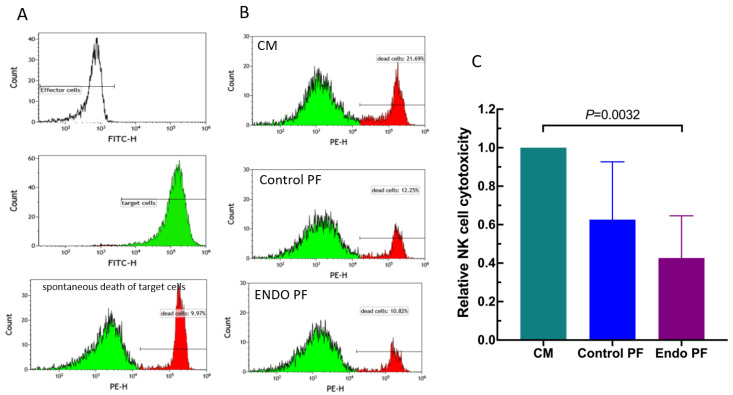
Effect of culture medium alone (CM), peritoneal fluid from control woman (Control PF) or peritoneal fluid from woman with endometriosis (ENDO PF) on the NK cell cytotoxic activity of cultured PBMC. (**A**) The representative flow cytometry analysis showing the controls for the NK assay. Shown are the fluorescence-negative effector cells (PBMC), green fluorescence (FITC) labeled K562 target cells and spontaneously dying K562 cells (red fluorescence, PE). Spontaneous death of target cells was determined in cultures without effector cells. (**B**) An example of identification of target K562 cells killed by NK cells from the PBMC population (red fluorescence). (**C**) Relative cell mediated cytotoxicity of untreated and peritoneal fluid-treated PBMC against K562 cells. The results are expressed as an index of specific cytotoxicity of peritoneal fluid-treated PBMC relative to untreated control PBMC. Each bar represents mean ± SD from 4 independent experiments. Statistical significance was computed by paired non-parametric ANOVA (Friedman’s test) followed by a post hoc test.

To reveal an effect of the PF from the patients with endometriosis and the control subjects on the NK cells, we evaluated the cytotoxic activity of the cultured PBMC against K562 erythroleukemia cells. As seen in Figure 5, a one day culture of PBMC with endometriotic PF resulted in a significant decrease in their cytotoxic activity. The control PF also displayed some inhibitory effect; this, however, was not statistically significant. There was also no significant difference between PF from endometriosis and control group.

## 3. Discussion

The results of the present study show for the first time that PF from women with advanced endometriosis displays immunomodulatory activity toward both unstimulated and CD3/CD28/IL-2-stimulated CD4^+^ T cells. We chose the stimulation of CD4^+^ T cells with CD3/CD28 beads and IL-2 as this method is considered to be a good model for assessment of T cell receptor-dependent T cell activation and expansion [27,28]. We found that endometriotic PF inhibited the production of IL-2, IFN-γ, IL-17A and TNF by CD4^+^ T cells. On the other hand, it stimulated the production of IL-4 and IL-10. The production of IL-2, IFN-γ and TNF is a feature of the Th1 subpopulation of CD4^+^ T cells, which is responsible for inflammatory and cell-mediated immunity, whereas the production of IL-4 and IL-10 is an attribute of Th2 cells, which are involved in the regulation of antibody production and the downregulation of cell-mediated responses [29,30]. Thus, our results may suggest that endometriotic PF displays an ability to shift CD4^+^ T cell differentiation into the Th2 phenotype. This observation is in line with the previous suggestions that the Th1/Th2 balance is abrogated in the endometriosis patients and that Th2 cells may favor development of the disease [31,32]. It should be stressed, however, that our observations were limited to cytokine evaluations and further studies on the shifting of the Th1/Th2 balance to Th2 phenotype are required.

We also found that the PF from women with endometriosis modulates the production of some T cell-derived chemokines. The treatment of CD4^+^ T cells with endometriotic PF resulted in stimulation of CCL2 (also known as MCP-1) release. This is consistent with our observation that CCL2 concentrations are elevated in the PF from endometriosis patients as compared to healthy women (Table 2) as well as previous observations of many other investigators [33]. CCL2 is a key chemokine responsible for chemotaxis/infiltration and activation of monocytes/macrophages [34], thus, our present results strongly argue for the role of the PF milieu in generation of pelvic inflammation in the course of endometriosis. Interestingly, we also found that endometriotic PF inhibited the production of CCL5 (RANTES) and CXCL9 (MIG) by the CD4^+^ T cells. CCL5 is responsible for the chemotaxis of T cells and some other leukocyte populations [35] and is considered to play a part in the pathogenesis of endometriosis [33]. CXCL9 is also responsible for T and NK cell infiltration, and, in particular, Th1 cells [36,37]. The expression of CXCL9 is upregulated by IFN-γ, thus, its downregulated production in endometriotic PF may reflect the inhibition of this cytokine release. These results seem to be in line with and extend the previous observations of Na et al. that endometriotic PF modulated production of CCL2, CCL3 (MIP-1α) and CCL5 by monocytes, neutrophils and T cells [38]. These findings strongly suggest that the PF from women with endometriosis displays immunosuppressive properties which may affect local infiltration and differentiation of T cells.

In addition to the observation that the PF from women with endometriosis affects cytokine/chemokine production by CD4^+^ T cells. We also found that it stimulates differentiation/expansion of CD25^high^ FOXP3^+^ Treg cells. Increased numbers of Treg cells were repeatedly reported in the peritoneum of patients with endometriosis, thus suggesting their role in the suppression of the local immune responses [17,23,25,39,40]. Our present result suggests that the mechanism responsible for the increased numbers of Treg cells in the endometriotic PF may be at least partially due to their local activation by the peritoneal milieu.

A stimulatory effect of the endometriotic PF on generation of Treg cells was accompanied by an inhibition of expansion of CD161^+^ Th17 cells. However, it should be noted that we did not observe any effect on the CD161^+^ RORg^+^ cells. It has been claimed that Th17 cells may play a part in the immunopathogenesis of endometriosis by exacerbation of the inflammatory response [41,42,43]. Nevertheless, our present observation suggests that the activity of Th17 cells may be suppressed by the factors present in the PF. Our observation might also explain the differential levels of Treg and Th17 cells in patients with different stage of the endometriosis [25].

Finally, we also confirmed the previous observations that the peritoneal fluid form women with endometriosis may also inhibit the cytotoxic activity of the NK cells [44,45].

Interestingly, the suppressive/modulatory effects of the PF of women with endometriosis were also reported on monocytes/macrophages. Accordingly, PF from the patients with endometriosis was reported to downregulate the expression of the MHC class II molecules as well as CD80 and CD86 costimulatory molecules in monocytes [46]. Furthermore, PF from women with endometriosis was also found to inhibit production of matrix metalloproteinases in the peritoneal macrophages [47].

Taken all together, our present results provide evidence that the PF of patients with endometriosis displays immunomodulatory/immunosuppressive activities toward CD4^+^ T cells. These activities manifest by inhibition of Th1 and stimulation of Th2 cytokine production, the inhibition of some lymphocyte chemotactic factor production, the shift of the Treg/Th17 balance to Treg phenotype and the inhibition of NK cell cytotoxic activity. The nature of these modulatory/suppressive properties of the endometriotic PF remains a subject of speculations. It should be stressed, however, that unlike in comparison to culture medium control, there were no significant differences in immunomodulatory/immunosuppressive activities between endometriotic PF and PF from control women without the disease. Furthermore, some inhibition of IL-2 and stimulation of IL-10 production by CD4^+^ T cells was also seen in the case of PF from the control women. The control PF also stimulated CCL2 and inhibited CCL5 and CXCL9 production. This strongly suggests that PF from control women with ovarian dermoid cysts also displays some immunoregulatory activity. We included patients with ovarian dermoid cysts as control since this is a benign ovarian teratoma that typically does not manifest with local inflammatory response or systemic immune deviations [48]. Considering that the immunology of ovarian dermoid cysts remains elusive, the significance our present observation and the nature of this phenomenon remains to be elucidated.

The levels of some investigated cytokines and chemokines, such as IL-6, IL-10, CCL2, CXCL8, and CXCL9, were significantly increased in PF from patients with endometriosis as compared to control. This observation is consistent with a variety of previous reports [20,21,49,50] and argues for the role of endometriotic peritoneal milieu in the regulation of local inflammatory responses. It is tempting to speculate that the modulatory/suppressive activity of the endometriotic PF is at least partially attributable to the increased local production of some regulatory cytokines such as TGF-β and IL-10 [20]. Both cytokines exert strong anti-inflammatory activity and were found to be produced by and to facilitate the induction of Treg cells [51,52,53,54]. TGF-β may be also responsible for local inhibition of the NK cell activity [45]. It should be stressed, however, that the regulation of the Treg/Th17 balance cannot simply be explained by an excessive stimulation with TGF-β [55] and this issue requires further investigations. Similarly, it is also difficult to speculate about the possible mechanisms responsible for the change of the Th1/Th2 balance. The differentiation of both subpopulations of Th cells appears to be a complicated phenomenon depending on a bulk of immunoregulatory cytokines and accessory cells [56] and also deserves further study.

It should be stressed that due to difficulties in obtaining the sufficient amounts of PF for the research purposes, the present study was performed on a limited number of PF samples. Nevertheless, the present results suggest that the peritoneal milieu of women with endometriosis shows immunosuppressive properties and shifts the Th1/Th2 balance toward the Th2 phenotype. These properties may help us to understand how the endometrioid tissue may escape from under local immune surveillance. The shift of the Th1/Th2 balance may also account for the dysregulated control of antibody production and may explain the origin of endometriosis-associated autoimmune phenomena. This may strongly support the view that the peritoneal milieu plays an important part in the pathogenesis of endometriosis and may be a target for specific clinical interventions.

## 4. Materials and Methods

### 4.1. Patients

The study included 16 women (mean age 35.8 years, range 25–46) with laparoscopically and histopathologically confirmed endometriosis. All patients had ovarian endometriotic cysts and the disease was classified as moderate/severe (III/IV) stage according to the revised criteria of the American Society for Reproductive Medicine [57]. The control group comprised 14 women (mean age 31.8 years, range 19–46) without visible endometriosis foci, pelvic inflammation or related pathology who underwent laparoscopic excision of ovarian dermoid cysts. All women had regular menses and none of them had a history of previous pelvic surgery or chronic systemic disease. The patients were not subjected to any hormonal or immunomodulatory therapy for at least six months prior to the study.

All participants gave a written informed consent to the study. The procedures were approved by the Institutional Bioethical Review Board of the Medical University of Warsaw, Poland and were conducted according to the Helsinki Declaration ethical principles.

### 4.2. PF Sample Collection

PF samples were collected on the same day at the mid-follicular phase (8–10 day) of the menstrual cycle. The mid-follicular menstrual cycle phase was additionally confirmed by the ultrasound examination.

PF was aspirated from the cul de sac at the beginning of the standard laparoscopic procedure under general anesthesia. Samples of the peritoneal fluid contaminated with blood were excluded from the study. PF samples were centrifuged at 400g at 4 °C for 10 min and the cell-free supernatants were collected, aliquoted, and stored frozen at −80 °C until used for further evaluations and experiments. The mean yield of PF obtained from the endometriosis patients and the control subjects was 6.2 (range 2–12.5) and 4.6 (range 0.5–13) mL, respectively.

### 4.3. Isolation, Stimulation and Culture of CD4^+^ T Cells

Peripheral blood mononuclear cells (PBMC) were isolated from the buffy coat from healthy volunteers from the local blood drive by Histopaque^®^-1077 (Sigma-Aldrich, St. Louis, MO, USA) density gradient centrifugation. Then, CD4^+^ T cells were isolated using CD4^+^ Cell Isolation Kit (Miltenyi Biotec, Bergisch Gladbach, Germany) according to the detailed protocol provided by the manufacturer. The purity of isolated cells was >90% as evaluated by flow cytometry analysis (see below). A representative flow cytometry analysis of CD4^+^ T cell purity is shown in Supplementary Figure S1.

Isolated CD4^+^ T cells were resuspended in RPMI 1640 culture medium supplemented with 10% Fetal Bovine Serum, 1% HEPES buffer and 1% Pen-Strep (all from Invitrogen, ThermoFisher Scientific, Waltham, MA, USA) and subjected or not to stimulation with Dynabeads™ Human T-Activator CD3/CD28 [27,28] at bead-to-cell ratio 1:1 and 30 U/mL rIL-2 (all from Invitrogen, TermoFisher Scientific) according to the protocol provided by the manufacturer. Then, 1 × 10^6^ of unstimulated or stimulated cells were cultured for 5 days in the medium alone or in the medium with control or endometriotic PF at 1:1 ratio without medium refreshment in the wells of 12-well plates (Corning Inc., Corning, NY, USA) at 37 °C and 5% CO_2_ atmosphere.

The following culture cell-free supernatants were collected and stored frozen at −70 °C until used for cytokine and chemokine quantification. The cells were also harvested, and their phenotype was evaluated by flow cytometry as described below.

Peritoneal fluids containing particular cytokines or chemokines at concentrations far exceeding interquartile range values were not used in the experiments. Baseline concentration ranges of each tested cytokines and chemokines present in control and endometriotic PF used in the experiments are given in the legends to Figure 2 and Figure 3. As seen, these baseline concentrations of cytokines and chemokines were relatively very low compared to those found in the cell-free supernatants following CD4^+^ T cell cultures and therefore may be considered as negligible.

### 4.4. Cytokine Evaluations

Concentrations of cytokines (IL-2, IL-4, IL-6, IL-10, IL-17A, IFN-γ, and TNF) and chemokines (CCL2, CCL5, CXCL8, and CXCL9) in peritoneal fluids and culture media were measured using the BD™ Cytometric Bead Array (CBA) Human Th1/Th2/Th17 Cytokine and Human Chemokine kits (BD Bioscience, USA), respectively. The samples were evaluated using a FACSVerse flow cytometry with BD Suite software (BD Bioscience) according to the protocol provided by the manufacturer. The results were analyzed with FCAP Array software (BD Bioscience). The advertised theoretical limit of detection defined as the corresponding concentration at two standard deviations above the median fluorescence of 20–30 replicates of the negative control (0 pg/mL) for IL-2, IL-4, IL-6, IL-10, IL-17A, IFN-γ, and TNF was 2.6, 4.9, 2.4, 4.5, 18.9, 3.7, and 3.8 pg/mL, respectively. The respective theoretical limit of detection for CCL2, CCL5, CXCL8, and CXCL9 was 2.7, 1.0, 0.2, and 2.5 pg/mL. The measurements were always within the respective standard curve. Raw data of standard curves for all assays are shown in a supplementary file.

### 4.5. Flow Cytometry Analysis

For flow cytometry analysis 0.5 × 10^6^ cells were labelled with 1 mg/mL of a respective antibody for 30 min at 4 °C, as described in detail elsewhere [23,58]. In brief, for evaluation of the purity of isolated CD4^+^ T cells the cells were labelled with FITC-conjugated anti-CD4 monoclonal antibodies (BD Biosciences, San Jose, CA, USA). For evaluation of Treg cells in CD4^+^ T cell cultures the harvested cells were labelled with PerCP-conjugated anti-CD4 and APC-conjugated anti-CD25 monoclonal antibodies (both from BD Biosciences) followed by a permeabilization-fixation procedure and intracellular staining with Phycoerythrin (PE) Anti-Human Foxp3 Staining Set (eBioscience Inc., San Diego, CA, USA) according to the detailed protocol provided by the manufacturer. For identification and evaluation of Th17 cells cultured CD4^+^ T cells were labelled with FITC-conjugated anti-CD161 monoclonal antibody (BD Biosciences) followed by intracellular staining with Phycoerythrin (PE)-conjugated Anti-Human ROR-γ antibody (eBioscience Inc.). As a negative control served nonspecific isotype IgG antibodies conjugated with the respective fluorochrome.

Cell samples were analyzed on the FACSCalibur using CellQuest / BD FACS Diva^TM^ software (BD Biosciences). The cells were specifically analyzed by selective gating, based on the parameters of forward and side scatter as described elsewhere [23,58]. The results were based on analysis of at least 100,000 cells and were shown as the percentage of positively labelled cells. The gating strategy for identification and evaluation of Treg and Th17 cells is shown in Supplementary Figures S2 and S3, respectively.

### 4.6. NK Cell Cytotoxicity Assay

PBMC were isolated from the buffy coat by Histopaque^®^-1077 (Sigma-Aldrich) density gradient centrifugation, washed and cultured in RPMI 1640 + GlutaMAX medium supplemented with 10% FBS and 1% antibiotic–antimycotic solution (all from Invitrogen, TermoFisher Scientific) with or without addition of PF (1:1) from patients with endometriosis or control subjects at a density of 2 × 10^6^/mL in 12-well plates at 37 °C in 5% CO_2_ atmosphere. Following 24 h of culture natural cytotoxic activity of PBMC was evaluated by means of NKTEST™ (Glycotope Biotechnology, Heidelberg, Germany) according to the detailed description provided by the manufacturer. In brief, cultured effector PBMC and K562 target cells prestained with a green fluorescent membrane dye were mixed at 50:1, 25:1 and 12.5:1 effector-to-target (E:T) ratio in a test medium and incubated for 3 h at 37°C in 5% CO_2_ atmosphere. Following incubation, the cells were stained with DNA staining solution for 5 min at 4 °C and the cytotoxicity was measured using CytoFLEX (Beckman Coulter) and CytExpert 2.0 software. Specific cytotoxicity was calculated on the basis of analysis of 5000 target cells and shown as percentage of positively stained cells. The results of cytotoxicity of PF-preincubated effector cells were presented in relation to control effector cells preincubated in medium alone.

### 4.7. Statistical Analyses

All statistical analyses and graphical presentations were performed using GraphPad Prism 8.2.0 (GraphPad Software, San Diego, CA, USA). Statistical differences between groups were calculated using the Mann–Whitney U-test or non-parametric analysis of variance (ANOVA) for paired or unpaired samples followed by post hoc multiple comparison test where applied. Differences were considered significant at least at *p* < 0.05.

## Figures and Tables

**Table 1:** Immunoregulatory cytokine concentrations in peritoneal fluid from patients with endometriosis and control women.

Cytokine	Control (n = 14)	Endometriosis (n = 16)	*p*-Value *
IL-2	0.3 (0.1–0.6)	0.4 (0.1–0.9)	ns
IFN-γ	0.3 (0–1.0)	0.1 (0–0.9)	ns
IL-17A	2.0 (0–8.1)	3.5 (0–28.4)	ns
TNF	1.2 (0.4–1.4)	0.9 (0.4–2.2)	ns
IL-4	0 (0–1.4)	0 (0–0.6)	ns
IL-10	1.5 (0–9.7)	8.0 (1.5–96.9)	0.0107
IL-6	35.8 (1.7–312.8)	313 (26.2–6436)	0.0435

Values are expressed in pg/mL and are shown as medians (range). * *p*-values were calculated by Mann–Whitney U test. ns, non-significant.

**Table 2:** Chemokine concentrations in peritoneal fluid from patients with endometriosis and control women.

Chemokine	Control (n = 14)	Endometriosis (n = 16)	*p*-Value *
CCL2	64.7 (16.6–198.7)	316.8 (10.1–10,333)	0.0232
CCL5	14.2 (5.8–227.2)	8.0 (5.3–33.9)	ns
CXCL8	7.7 (1.4–62.0)	72.2 (8.7–9027)	0.0015
CXCL9	16.6 (5.2–50.8)	46.2 (24.1–628.3)	0.0030

Values are expressed in pg/mL and are shown as medians and a range. * *p*-values were calculated by Mann–Whitney U test. ns, non-significant.

## Data Availability

Raw data can be obtained from the corresponding author upon request.

## Supplementary Materials

The following are available online at https://www.mdpi.com/article/10.3390/ijms22158134/s1.

## References

[1] Zondervan K.T., Becker C.M., Missmer S.A. (2020). Endometriosis. N. Engl. J. Med..

[2] Koninckx P.R., Ussia A., Adamyan L., Wattiez A., Gomel V., Martin D.C. (2019). Pathogenesis of endometriosis: The genetic/epigenetic theory. Fertil. Steril..

[3] Giudice L.C., Kao L.C. (2004). Endometriosis. Lancet.

[4] Tomassetti C., D’Hooghe T. (2018). Endometriosis and infertility: Insights into the causal link and management strategies. Best Pract. Res. Clin. Obstet. Gynaecol..

[5] Nisolle M., Donnez J. (1997). Peritoneal endometriosis, ovarian endometriosis, and adenomyotic nodules of the rectovaginal septum are three different entities. Fertil. Steril..

[6] Garcia-Velasco J.A., Arici A. (2003). Apoptosis and the pathogenesis of endometriosis. Semin. Reprod Med..

[7] Balkowiec M., Maksym R.B., Wlodarski P.K. (2018). The bimodal role of matrix metalloproteinases and their inhibitors in etiology and pathogenesis of endometriosis (Review). Mol. Med. Rep..

[8] Witz C.A. (2003). Cell adhesion molecules and endometriosis. Semin. Reprod. Med..

[9] Reis F.M., Petraglia F., Taylor R.N. (2013). Endometriosis: Hormone regulation and clinical consequences of chemotaxis and apoptosis. Hum. Reprod Update.

[10] Sciezynska A., Komorowski M., Soszynska M., Malejczyk J. (2019). NK Cells as Potential Targets for Immunotherapy in Endometriosis. J. Clin. Med..

[11] Matarese G., De Placido G., Nikas Y., Alviggi C. (2003). Pathogenesis of endometriosis: Natural immunity dysfunction or autoimmune disease?. Trends Mol. Med..

[12] Zhang T., De Carolis C., Man G.C.W., Wang C.C. (2018). The link between immunity, autoimmunity and endometriosis: A literature update. Autoimmun. Rev..

[13] Riccio L., Santulli P., Marcellin L., Abrao M.S., Batteux F., Chapron C. (2018). Immunology of endometriosis. Best Pract. Res. Clin. Obstet Gynaecol..

[14] Eisenberg V.H., Zolti M., Soriano D. (2012). Is there an association between autoimmunity and endometriosis?. Autoimmun. Rev..

[15] Berbic M., Fraser I.S. (2011). Regulatory T cells and other leukocytes in the pathogenesis of endometriosis. J. Reprod. Immunol..

[16] Ulukus M., Arici A. (2005). Immunology of endometriosis. Minerva Ginecol..

[17] de Barros I.B.L., Malvezzi H., Gueuvoghlanian-Silva B.Y., Piccinato C.A., Rizzo L.V., Podgaec S. (2017). What do we know about regulatory T cells and endometriosis? A systematic review. J. Reprod. Immunol..

[18] Vallve-Juanico J., Houshdaran S., Giudice L.C. (2019). The endometrial immune environment of women with endometriosis. Hum. Reprod Update.

[19] Izumi G., Koga K., Takamura M., Makabe T., Satake E., Takeuchi A., Taguchi A., Urata Y., Fujii T., Osuga Y. (2018). Involvement of immune cells in the pathogenesis of endometriosis. J. Obstet. Gynaecol. Res..

[20] Zhou W.J., Yang H.L., Shao J., Mei J., Chang K.K., Zhu R., Li M.Q. (2019). Anti-inflammatory cytokines in endometriosis. Cell Mol. Life Sci..

[21] Gazvani R., Templeton A. (2002). Peritoneal environment, cytokines and angiogenesis in the pathophysiology of endometriosis. Reproduction.

[22] Basta P., Majka M., Jozwicki W., Lukaszewska E., Knafel A., Grabiec M., Stasienko E., Wicherek L. (2010). The frequency of CD25+CD4+ and FOXP3+ regulatory T cells in ectopic endometrium and ectopic decidua. Reprod. Biol. Endocrinol..

[23] Olkowska-Truchanowicz J., Bocian K., Maksym R.B., Bialoszewska A., Wlodarczyk D., Baranowski W., Zabek J., Korczak-Kowalska G., Malejczyk J. (2013). CD4(+) CD25(+) FOXP3(+) regulatory T cells in peripheral blood and peritoneal fluid of patients with endometriosis. Hum. Reprod..

[24] Podgaec S., Rizzo L.V., Fernandes L.F., Baracat E.C., Abrao M.S. (2012). CD4(+) CD25(high) Foxp3(+) cells increased in the peritoneal fluid of patients with endometriosis. Am. J. Reprod. Immunol..

[25] Khan K.N., Yamamoto K., Fujishita A., Muto H., Koshiba A., Kuroboshi H., Saito S., Teramukai S., Nakashima M., Kitawaki J. (2019). Differential levels of regulatory T-cells and T-helper-17 cells in women with early and advanced endometriosis. J. Clin. Endocrinol. Metab..

[26] Sikora J., Smycz-Kubanska M., Mielczarek-Palacz A., Bednarek I., Kondera-Anasz Z. (2018). The involvement of multifunctional TGF-beta and related cytokines in pathogenesis of endometriosis. Immunol. Lett..

[27] Trickett A., Kwan Y.L. (2003). T cell stimulation and expansion using anti-CD3/CD28 beads. J. Immunol. Methods.

[28] Martkamchan S., Onlamoon N., Wang S., Pattanapanyasat K., Ammaranond P. (2016). The Effects of Anti-CD3/CD28 Coated Beads and IL-2 on Expanded T Cell for Immunotherapy. Adv. Clin. Exp. Med..

[29] Romagnani S. (2000). T-cell subsets (Th1 versus Th2). Ann. Allergy Asthma Immunol..

[30] Hirahara K., Nakayama T. (2016). CD4+ T-cell subsets in inflammatory diseases: Beyond the Th1/Th2 paradigm. Int. Immunol..

[31] Antsiferova Y.S., Sotnikova N.Y., Posiseeva L.V., Shor A.L. (2005). Changes in the T-helper cytokine profile and in lymphocyte activation at the systemic and local levels in women with endometriosis. Fertil. Steril..

[32] Podgaec S., Abrao M.S., Dias J.A., Rizzo L.V., de Oliveira R.M., Baracat E.C. (2007). Endometriosis: An inflammatory disease with a Th2 immune response component. Hum. Reprod..

[33] Borrelli G.M., Carvalho K.I., Kallas E.G., Mechsner S., Baracat E.C., Abrao M.S. (2013). Chemokines in the pathogenesis of endometriosis and infertility. J. Reprod. Immunol..

[34] Deshmane S.L., Kremlev S., Amini S., Sawaya B.E. (2009). Monocyte chemoattractant protein-1 (MCP-1): An overview. J. Interferon Cytokine Res..

[35] Marques R.E., Guabiraba R., Russo R.C., Teixeira M.M. (2013). Targeting CCL5 in inflammation. Expert Opin. Ther. Targets.

[36] Tokunaga R., Zhang W., Naseem M., Puccini A., Berger M.D., Soni S., McSkane M., Baba H., Lenz H.J. (2018). CXCL9, CXCL10, CXCL11/CXCR3 axis for immune activation—A target for novel cancer therapy. Cancer Treat Rev..

[37] Neo S.Y., Lundqvist A. (2020). The Multifaceted Roles of CXCL9 Within the Tumor Microenvironment. Adv. Exp. Med. Biol..

[38] Na Y.J., Lee D.H., Kim S.C., Joo J.K., Wang J.W., Jin J.O., Kwak J.Y., Lee K.S. (2011). Effects of peritoneal fluid from endometriosis patients on the release of monocyte-specific chemokines by leukocytes. Arch. Gynecol. Obstet..

[39] Berbic M., Hey-Cunningham A.J., Ng C., Tokushige N., Ganewatta S., Markham R., Russell P., Fraser I.S. (2010). The role of Foxp3+ regulatory T-cells in endometriosis: A potential controlling mechanism for a complex, chronic immunological condition. Hum. Reprod..

[40] Braundmeier A., Jackson K., Hastings J., Koehler J., Nowak R., Fazleabas A. (2012). Induction of endometriosis alters the peripheral and endometrial regulatory T cell population in the non-human primate. Hum. Reprod..

[41] Chang K.K., Liu L.B., Jin L.P., Zhang B., Mei J., Li H., Wei C.Y., Zhou W.J., Zhu X.Y., Shao J. (2017). IL-27 triggers IL-10 production in Th17 cells via a c-Maf/RORgammat/Blimp-1 signal to promote the progression of endometriosis. Cell Death Dis..

[42] Gogacz M., Winkler I., Bojarska-Junak A., Tabarkiewicz J., Semczuk A., Rechberger T., Adamiak A. (2016). Increased percentage of Th17 cells in peritoneal fluid is associated with severity of endometriosis. J. Reprod. Immunol..

[43] Hirata T., Osuga Y., Hamasaki K., Yoshino O., Ito M., Hasegawa A., Takemura Y., Hirota Y., Nose E., Morimoto C. (2008). Interleukin (IL)-17A stimulates IL-8 secretion, cyclooxygensase-2 expression, and cell proliferation of endometriotic stromal cells. Endocrinology.

[44] Oosterlynck D.J., Meuleman C., Waer M., Koninckx P.R., Vandeputte M. (1993). Immunosuppressive activity of peritoneal fluid in women with endometriosis. Obstet. Gynecol..

[45] Guo S.W., Du Y., Liu X. (2016). Platelet-derived TGF-beta1 mediates the down-modulation of NKG2D expression and may be responsible for impaired natural killer (NK) cytotoxicity in women with endometriosis. Hum. Reprod..

[46] Lee K.S., Baek D.W., Kim K.H., Shin B.S., Lee D.H., Kim J.W., Hong Y.S., Bae Y.S., Kwak J.Y. (2005). IL-10-dependent down-regulation of MHC class II expression level on monocytes by peritoneal fluid from endometriosis patients. Int. Immunopharmacol..

[47] Wu M.H., Shoji Y., Wu M.C., Chuang P.C., Lin C.C., Huang M.F., Tsai S.J. (2005). Suppression of matrix metalloproteinase-9 by prostaglandin E(2) in peritoneal macrophage is associated with severity of endometriosis. Am. J. Pathol..

[48] Ahmed A., Lotfollahzadeh S. (2021). Cystic Teratoma. Treasure Island.

[49] Barcz E., Milewski L., Dziunycz P., Kaminski P., Ploski R., Malejczyk J. (2012). Peritoneal cytokines and adhesion formation in endometriosis: An inverse association with vascular endothelial growth factor concentration. Fertil. Steril..

[50] Milewski L., Dziunycz P., Barcz E., Radomski D., Roszkowski P.I., Korczak-Kowalska G., Kaminski P., Malejczyk J. (2011). Increased levels of human neutrophil peptides 1, 2, and 3 in peritoneal fluid of patients with endometriosis: Association with neutrophils, T cells and IL-8. J. Reprod. Immunol..

[51] Kanamori M., Nakatsukasa H., Okada M., Lu Q., Yoshimura A. (2016). Induced Regulatory T Cells: Their Development, Stability, and Applications. Trends Immunol..

[52] Sanjabi S., Zenewicz L.A., Kamanaka M., Flavell R.A. (2009). Anti-inflammatory and pro-inflammatory roles of TGF-beta, IL-10, and IL-22 in immunity and autoimmunity. Curr. Opin. Pharmacol..

[53] Li M.O., Flavell R.A. (2008). TGF-beta: A master of all T cell trades. Cell.

[54] Li M.O., Flavell R.A. (2008). Contextual regulation of inflammation: A duet by transforming growth factor-beta and interleukin-10. Immunity.

[55] Lee G.R. (2018). The Balance of Th17 versus Treg Cells in Autoimmunity. Int. J. Mol. Sci..

[56] Zhang Y., Zhang Y., Gu W., Sun B. (2014). TH1/TH2 cell differentiation and molecular signals. Adv. Exp. Med. Biol..

[57] ASRM (1997). Revised American Society for Reproductive Medicine classification of endometriosis: 1996. Fertil. Steril..

[58] Bocian K., Borysowski J., Wierzbicki P., Wyzgal J., Klosowska D., Bialoszewska A., Paczek L., Gorski A., Korczak-Kowalska G. (2010). Rapamycin, unlike cyclosporine A, enhances suppressive functions of in vitro-induced CD4+CD25+ Tregs. Nephrol. Dial. Transplant.

