# Supplementary material for: Peritoneal Fluid from Patients with Ovarian Endometriosis Displays Immunosuppressive Potential and Stimulates Th2 Response
Source: Int J Mol Sci. 2021 Jul 29;22(15):8134. doi: 10.3390/ijms22158134 (PMC8347337; doi:10.3390/ijms22158134)
Supplement: Supplementary file 1 [file ijms-22-08134-s001.zip › ijms-1251724-supplementary.pdf]

SUPPLEMENTARY FIGURES

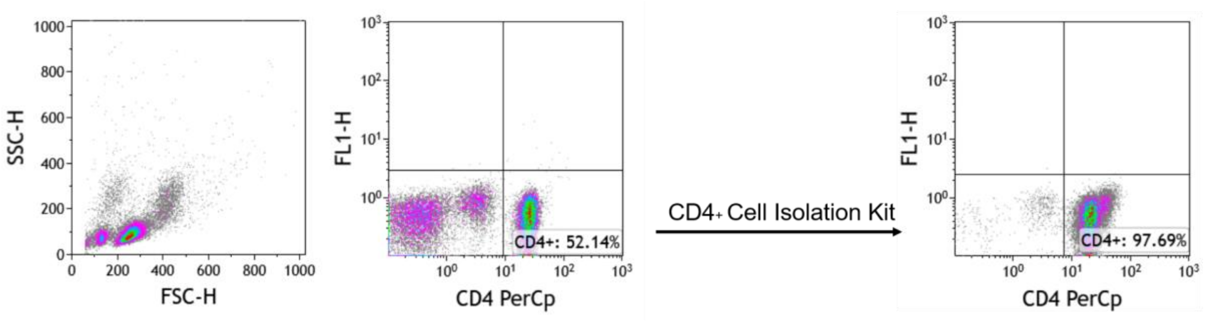

Supplementary Figure S1. A representative flow cytometry analysis of the purity of CD4$^+$ T cell populations obtained following isolation using magnetic beads CD4$^+$ cell isolation kit.

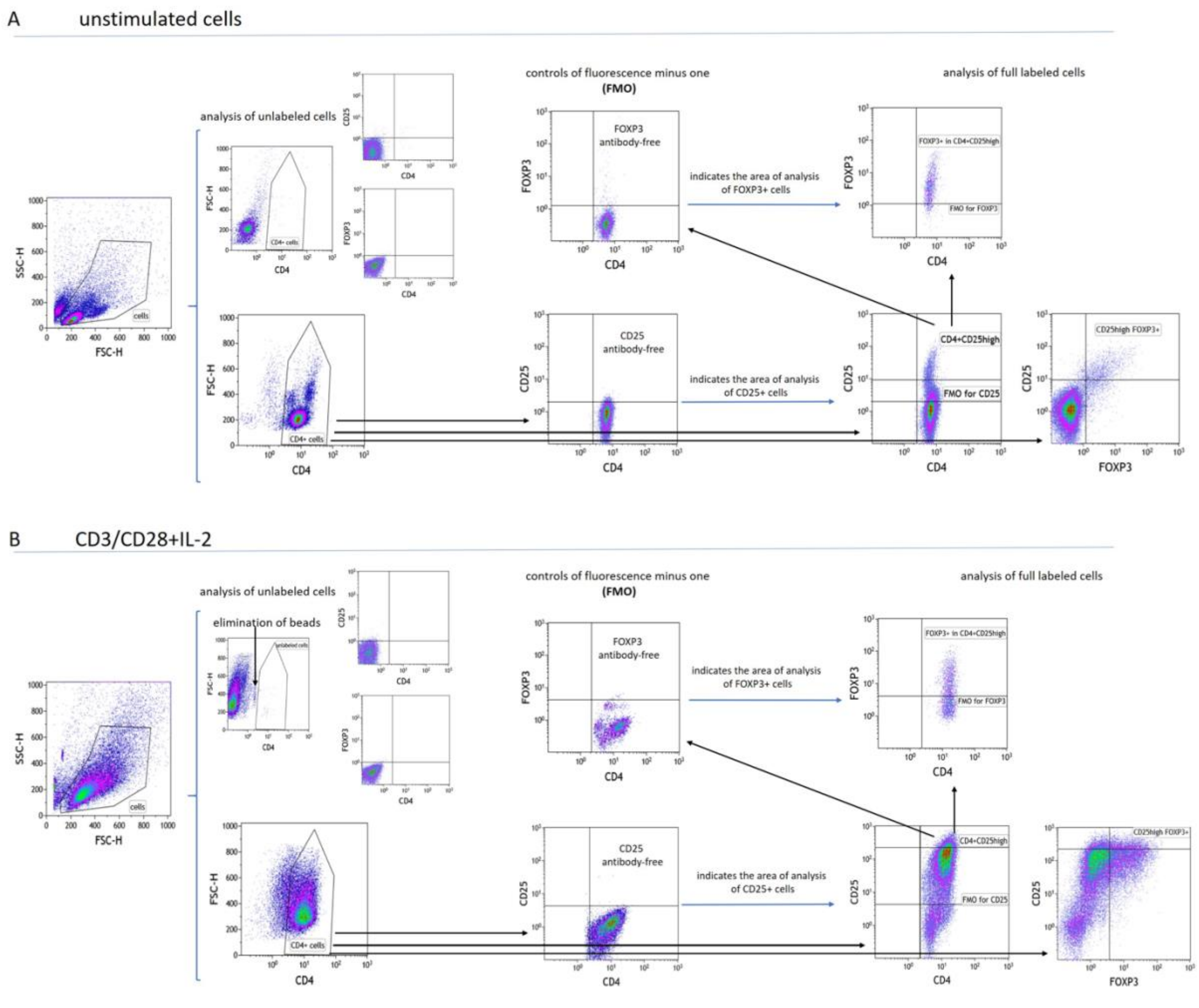

Supplementary Figure S2. Gating strategy for flow cytometry identification and evaluation of CD25^{high} and CD25^{high} FOXP3$^+$ Treg cells in (**A**) unstimulated control and (**B**) CD3/CD28 beads+IL-2-stimulated cultures.

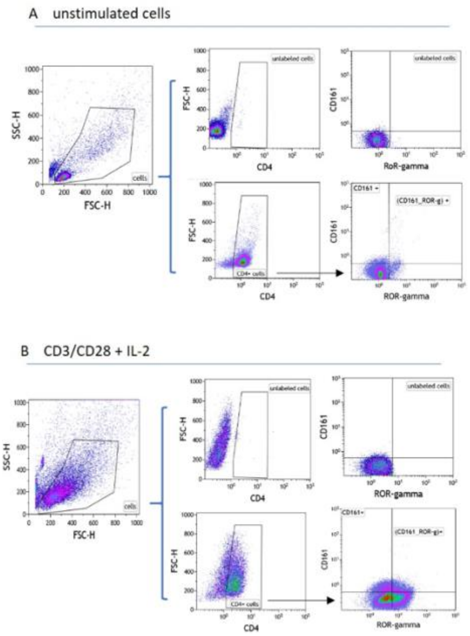

Supplementary Figure S3. Gating strategy for flow cytometry identification and evaluation of CD161^{+} and CD161^{+} RORγ^{+} Th17 cells in (**A**) unstimulated control and (**B**) CD3/CD28 beads+IL-2-stimulated cultures.
